# Supplementary material for: Differential active site requirements for NDM-1 β-lactamase hydrolysis of carbapenem versus penicillin and cephalosporin antibiotics
Source: Nat Commun. 2018 Oct 30;9:4524. doi: 10.1038/s41467-018-06839-1 (PMC6207675; doi:10.1038/s41467-018-06839-1)
Supplement: Supplementary file 1 — Supplementary Information [file 41467_2018_6839_MOESM1_ESM.pdf]

Supplementary Figure and Tables

Differential active site requirements for NDM-1 β-Lactamase hydrolysis of carbapenem versus penicillin and cephalosporin antibiotics

Sun *et al.*

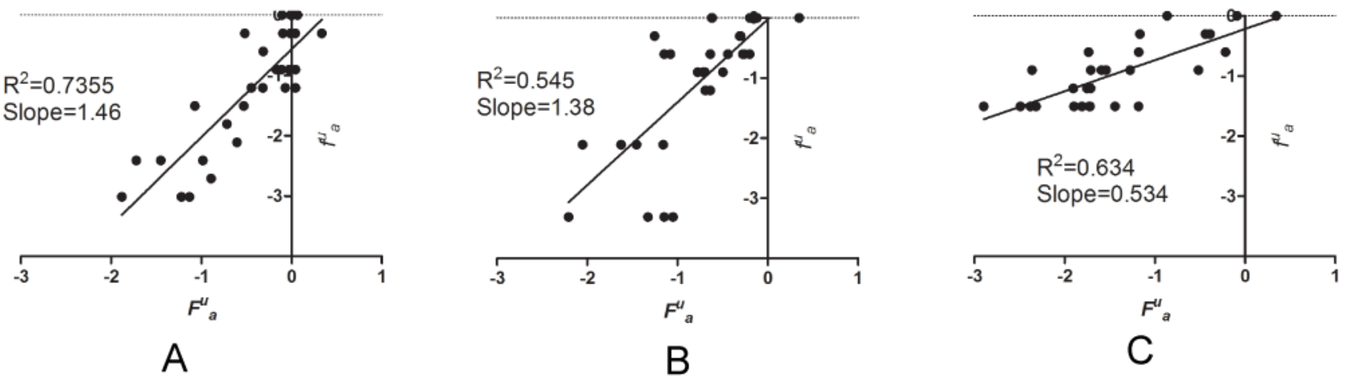

Supplementary Figure 1. Relative fitness of NDM-1 mutants based on sequencing versus antibiotic resistance levels. The relative fitness of individual NDM-1 mutants from the antibiotic selected libraries (F_u^a) was calculated based on the frequency of occurrence of the mutant and wild-type alleles from the deep sequencing data for the naïve library and antibiotic-selected libraries as described in Methods. The relative fitness of the mutants based on antibiotic resistance levels (f_u^a) was calculated as the logarithm of the resistance level of mutants relative to that of wild type for each antibiotic. The f_u^a value of each mutant is plotted versus F_u^a of the corresponding mutant for ampicillin (A), cefotaxime (B) and imipenem (C) experiments and the results of linear regression analysis are shown indicating a correlation between relative fitness determined based on sequencing data versus that determined based on antibiotic resistance levels of individual mutants.

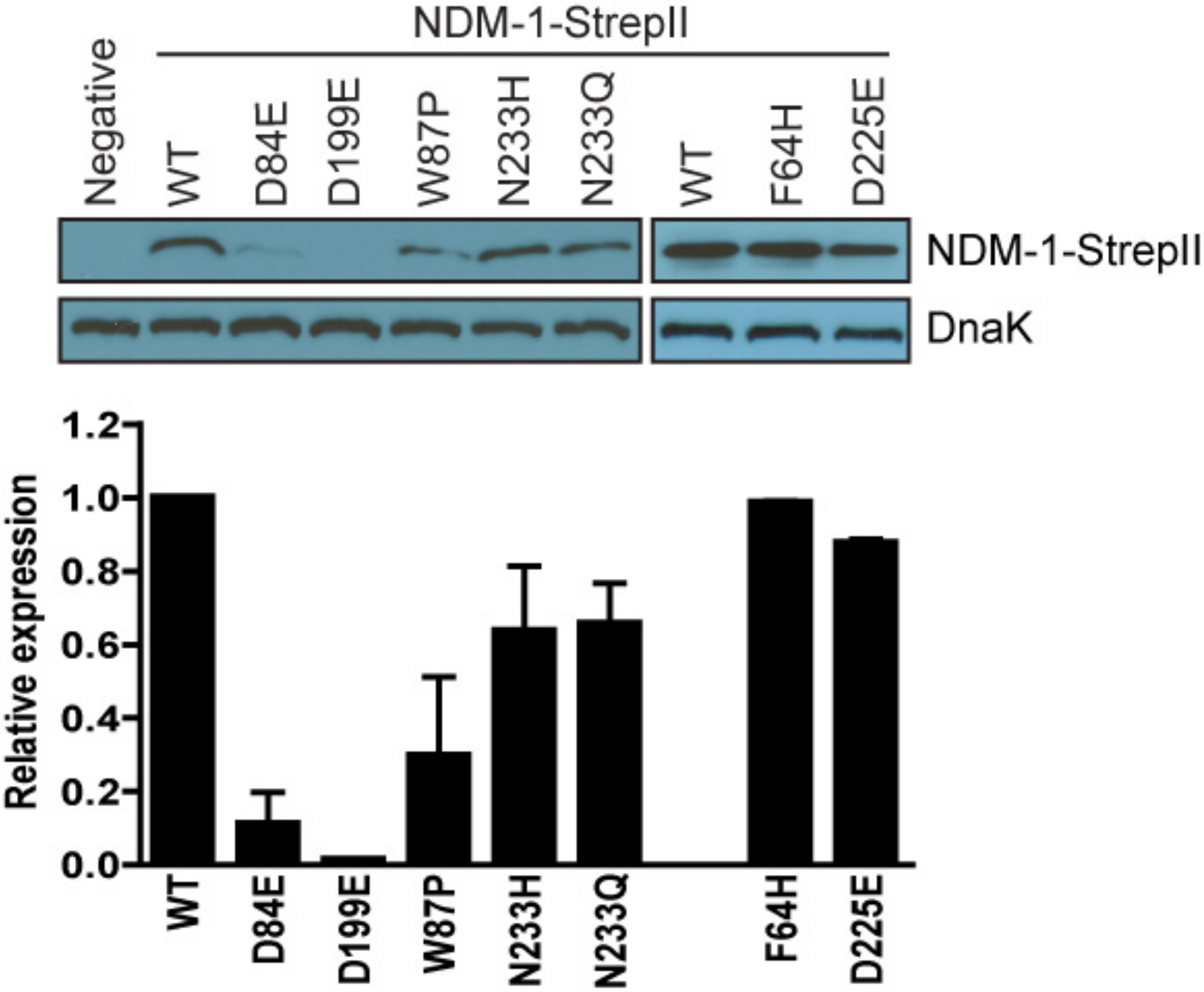

Supplementary Figure 2. *In vivo* steady-state protein levels of NDM-1 wild-type and mutants. Steady-state protein expression levels in *E. coli* of StrepII-tagged wild-type NDM-1 and mutants were determined by SDS-PAGE of whole cell lysates followed by immunoblotting with an anti-StrepII tag monoclonal antibody conjugated to horseradish peroxidase (HRP). Constitutively expressed DnaK (~70 kDa) was used as a loading control and probed with anti-DnaK monoclonal antibody and an HRP-conjugated secondary antibody. The hybridization signal for wild-type and mutant StrepII-tagged NDM-1 and DnaK was quantified by densitometry. The signal for NDM-1 was normalized to that for DnaK in the same sample. Protein expression levels of NDM-1 mutants are shown in the bar graph relative to that of wild-type NDM-1, which was set as 1. Quantification data are based on three independent experiments and a representative blot is shown. The error bars indicate the standard deviation of the protein expression levels based on the repeated experiments.

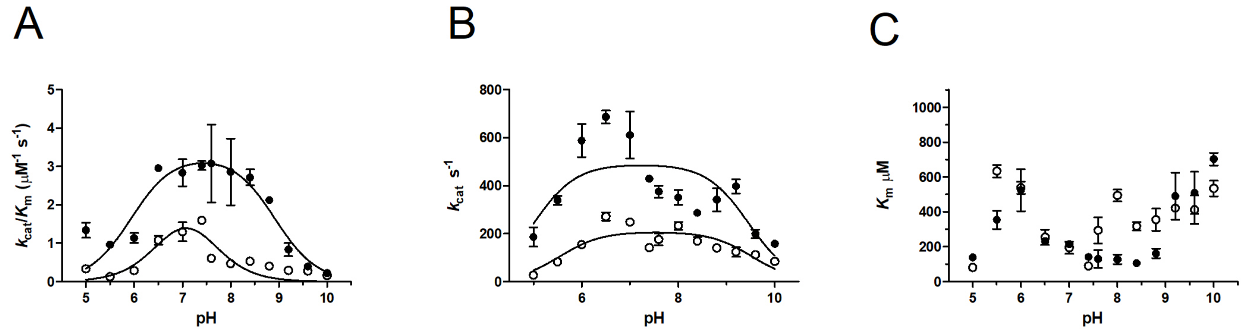

Supplementary Figure 3. pH profile for wild-type NDM-1 and the K224R/G232A/N233Q triple mutant. The pH dependence of k_{cat}/K_M (A), k_{cat} (B), K_M (C) of the wild type (closed circles) and the K224R/G232A/N233Q triple mutant (open circles) NDM-1 β-lactamase for ampicillin hydrolysis is shown. The error bars in the plots represent the standard deviations for each data point. Lys224 plays an important role in facilitating substrate binding to the active site through electrostatically interacting with the negatively charged C-3/4 carboxylate group of β-lactam antibiotics. Its substitution by arginine, which also has a positively charged side chain but with a different pKa (12.5) from that of lysine (10.5), may change the pH dependence of the charge status of their side chain groups and thus their interactions with β-lactam substrates. To test this hypothesis, the pH dependence of the wild type and K224R/G232A/N233Q mutant enzymes was examined for the hydrolysis of ampicillin. Fitting of the k_{cat} and k_{cat}/K_M to a double ionization model produced bell-shaped curves for k_{cat}/K_M of both wild type and the mutant enzyme. Although the optimum pH for k_{cat}/K_M is similar for wild type and mutant enzymes, the mutant enzyme exhibited a narrower pH profile (pK1 = 6.7 and pK2 = 7.5) than the wild-type enzyme (pK1 = 5.9 and pK2 = 8.9). This difference is mainly attributed to the larger fluctuation of the K_M of the mutant enzyme than that of the wild-type enzyme at pH 5.5-8.5. This indicates that complex formation with ampicillin is more sensitive to pH variation versus the wild-type enzyme.

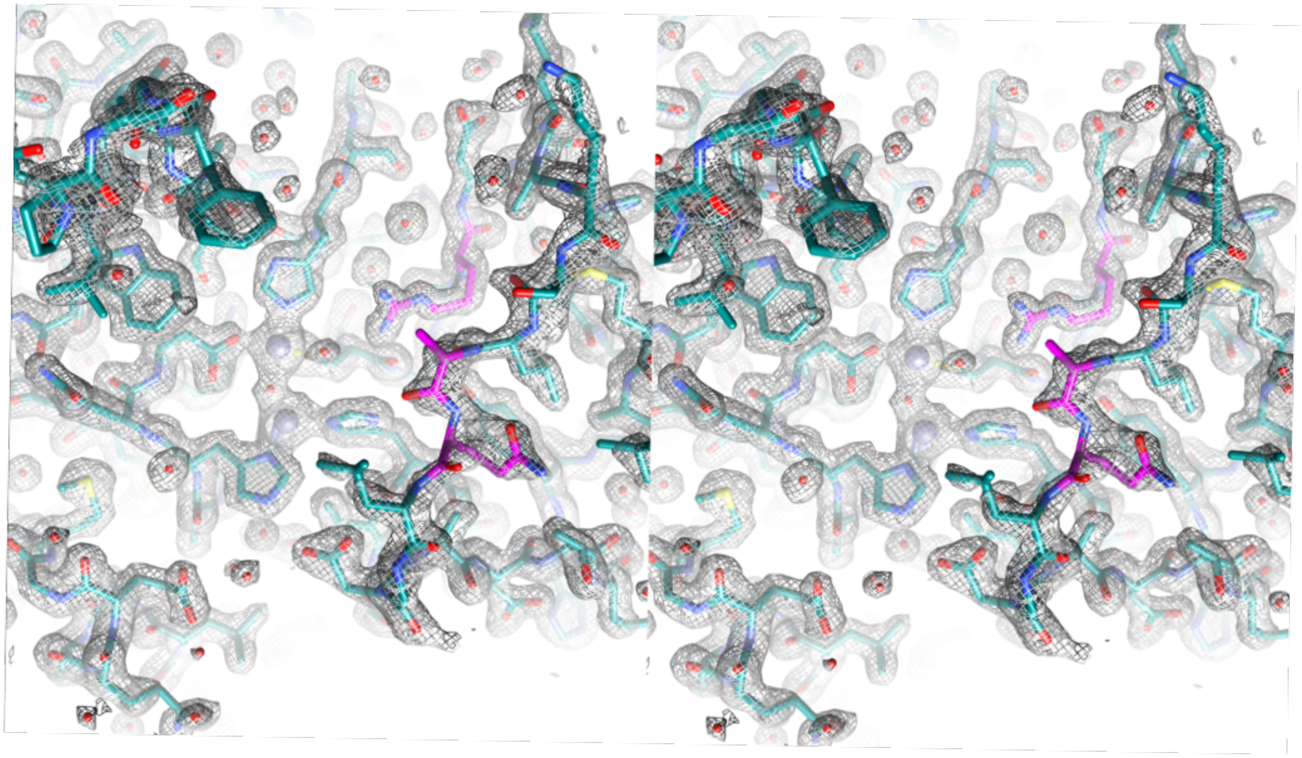

Supplementary Figure 4. Stereo view of the active site of the NDM-1 K224R/G232A/N233Q β-lactamase structure. Electron density is shown of an 2Fo-Fc map contoured at 1 σ. Zn1 and Zn2 are shown as gray spheres with Zn1 at top. Residues are represented as stick models with carbons colored dark cyan and non-carbon colored by type (N, blue; O, red; S, yellow). The mutated residues, Arg224, Ala232, and Gln233, are shown with carbon atoms in magenta.

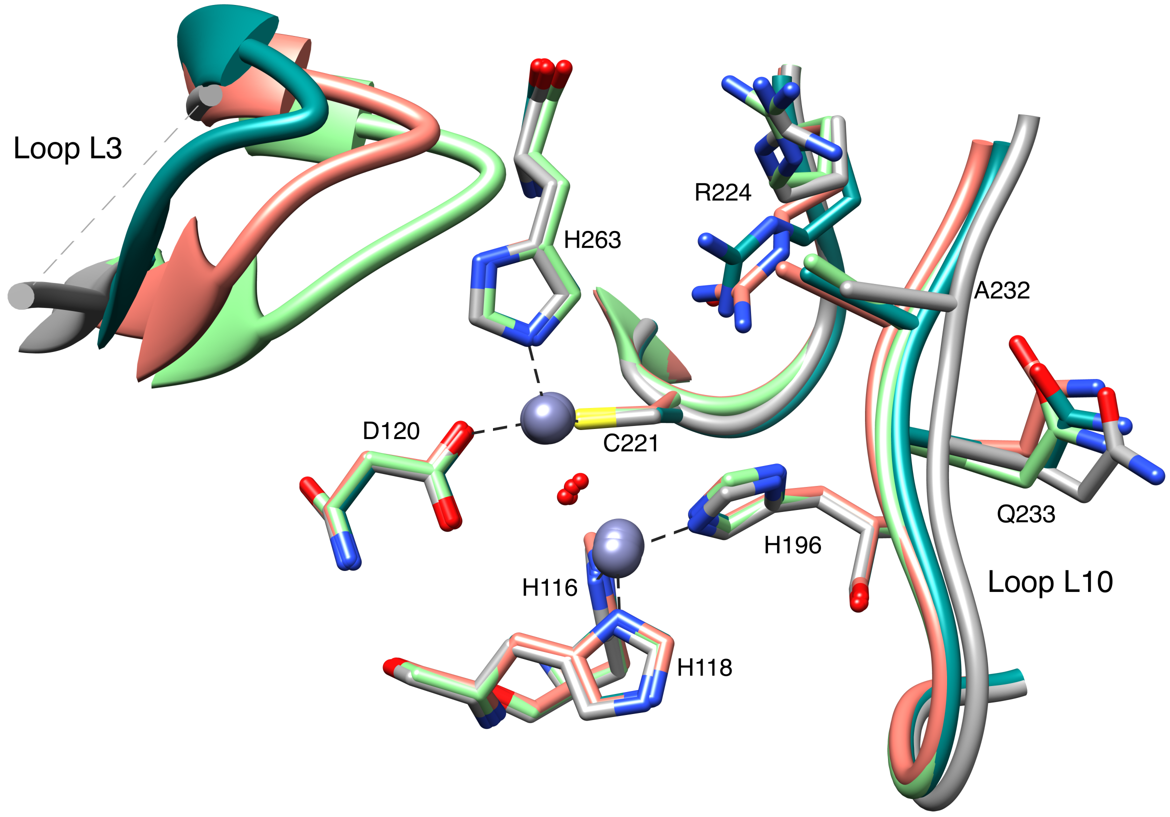

Supplementary Figure 5. Overlay of the four chains of the NDM-1 K224R/G232A/N233Q structure. Chains A, B, C, and D are represented as dark cyan, gray, pale green and salmon, respectively. Zinc ions are represented as gray spheres and the water molecule coordinated by the corresponding zinc ions is shown as a red sphere. Zinc-chelating residues (His116, His118, Asp120, His196, and His263), as well as residues Arg224, Ala232, and Gln233 are represented as stick model in all structures with non-carbon atoms colored by type (N, blue; O, red; S, yellow). The active site loop structures L3 and L10 are labeled.

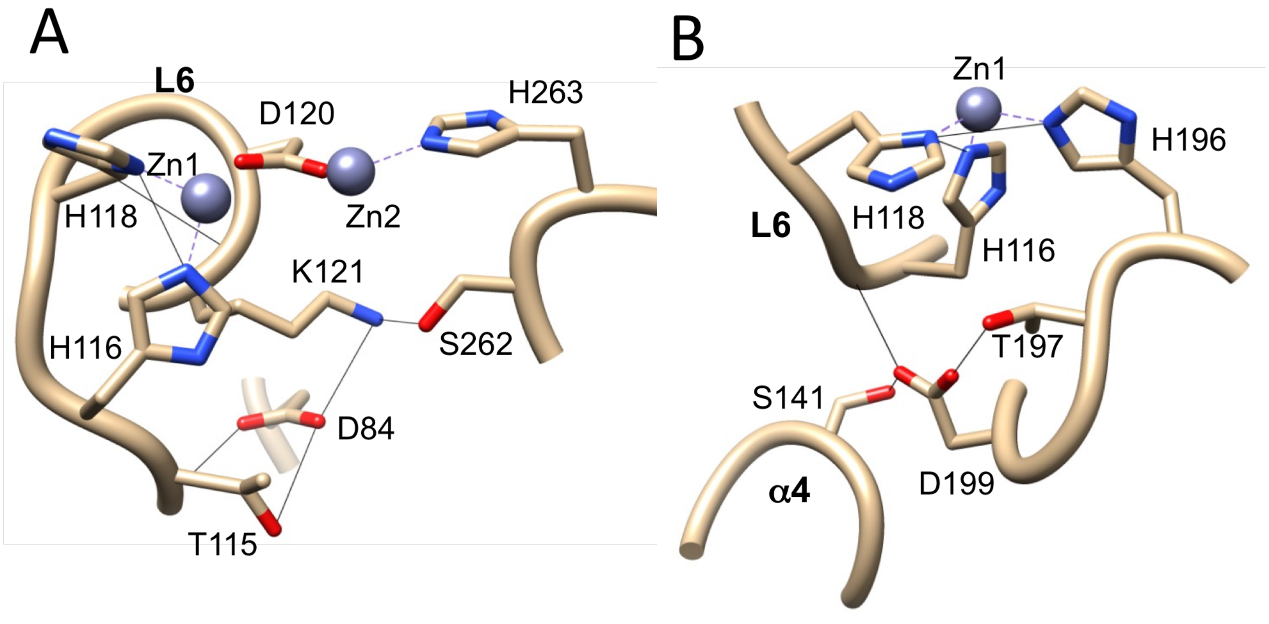

Supplementary Figure 6. Hydrogen bond networks linking loops in the NDM-1 active site. **A**. The network of interactions involving Asp84 and Lys121 is shwon. Note that, for clarity, not all zinc ligand residues are shown. **B**. The network of interactions involving Asp199 is shown. Hydrogen bonds are depicted as black lines. Black lines ending at the cartoon tube represent hydrogen bonds to the main chain CO or NH groups. Zinc ions are colored gray and labeled. Carbon is shown in tan, nitrogen is blue and oxygen is red. Side chains of residues are shown and the main chain is shown in cartoon tube.

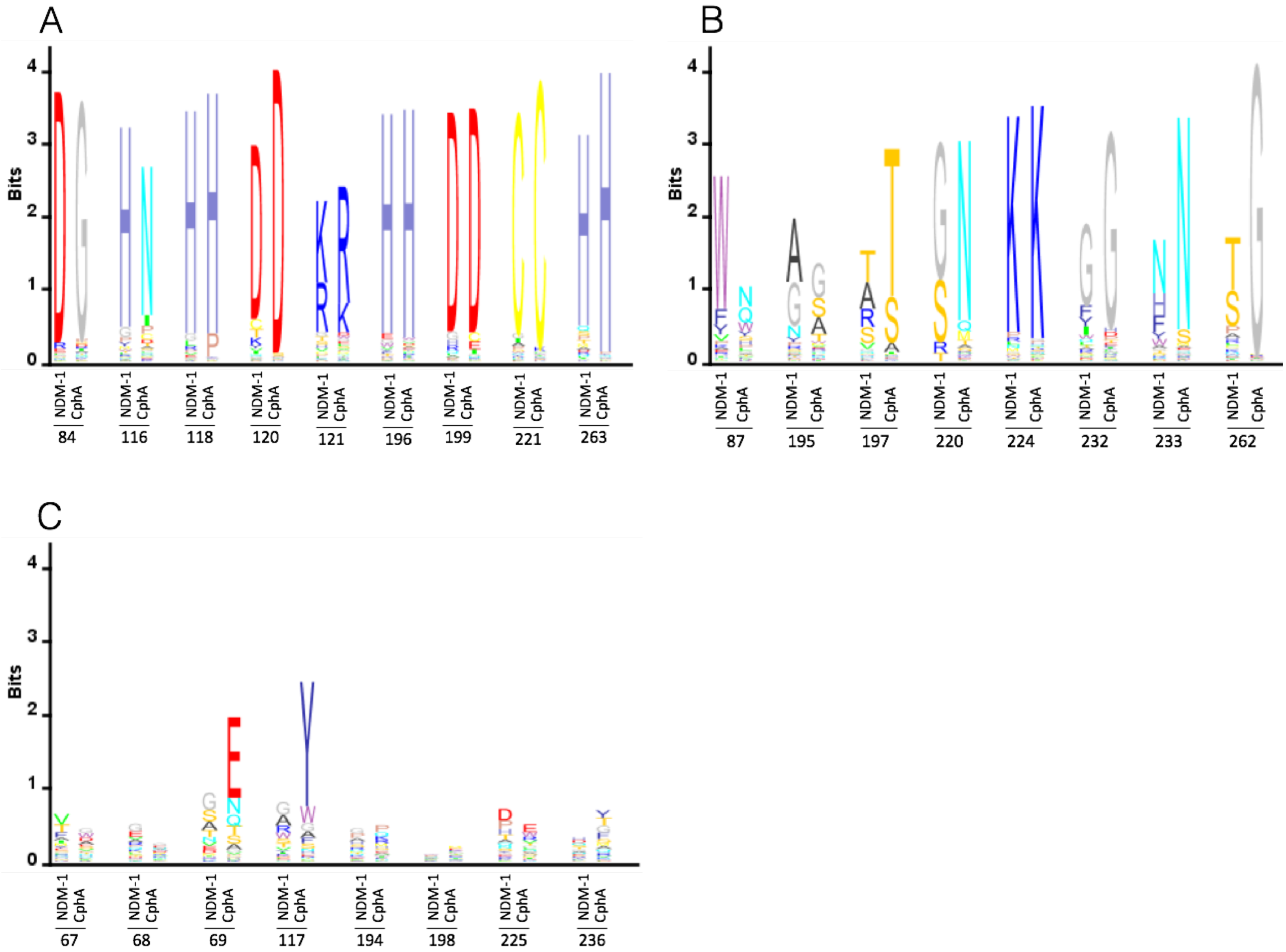

Supplementary Figure 7. Sequence logo comparison of the deep sequencing results for NDM-1 versus CphA β-lactamases. Deep sequencing results from single codon random libraries selected for imipenem resistance for the NDM-1 and CphA metallo-β-lactamases are compared using sequence logos. **A**. Essential residues for both enzymes. The wild-type residues dominate among sequences from populations selected for imipenem resistance. **B**. Residue positions where the sequence requirements for impenem resistance differ between NDM-1 and CphA β-lactamases. **C**. Non-essential residues, no specific residues strongly dominate after the imipenem resistance selection by either NDM-1 or CphA β-lactamase. The data for CphA is from reference [1].

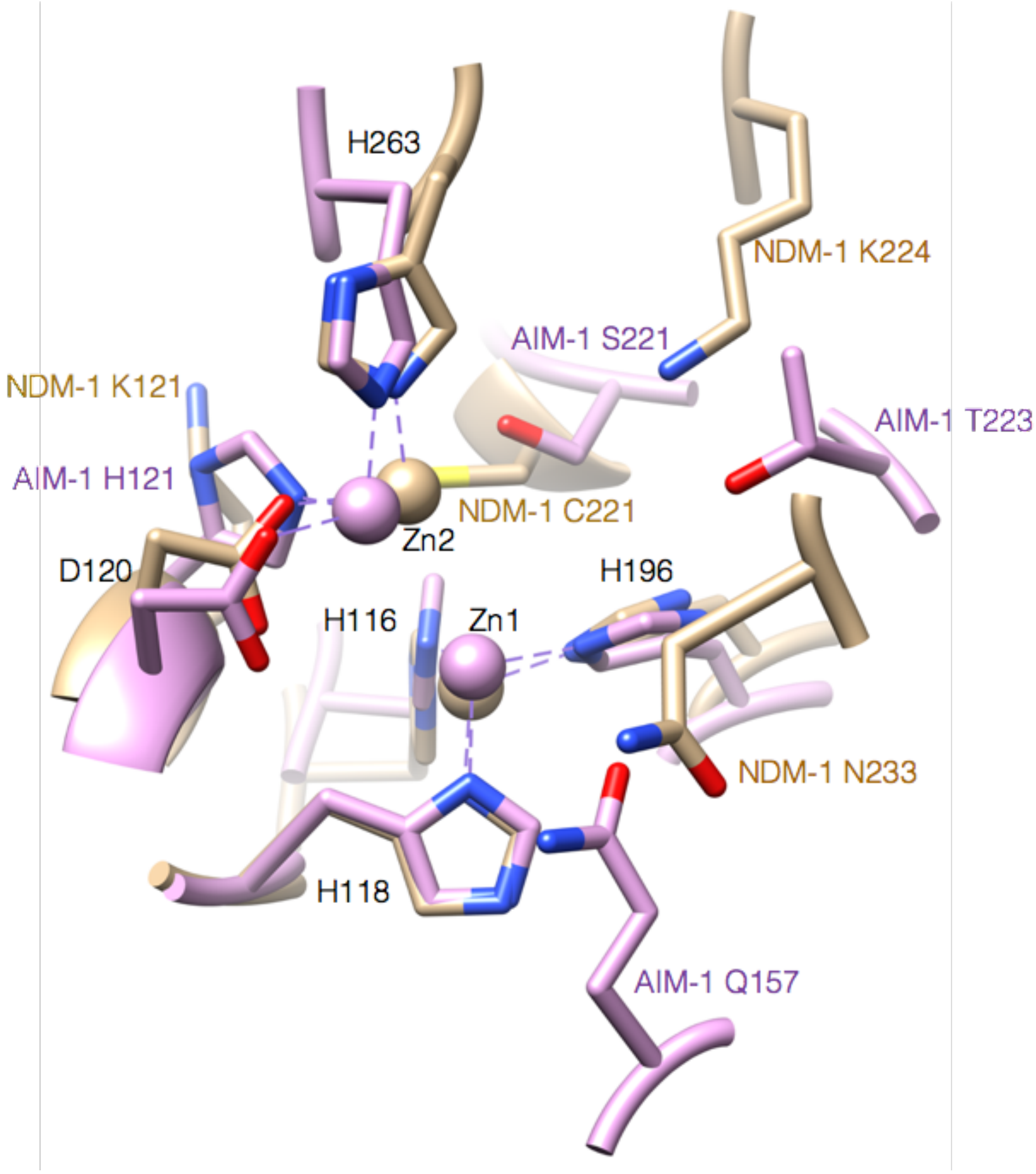

Supplementary Figure 8. Structure alignment of active site residues of subclass B1 NDM-1 (PDB ID: 3SPU)(tan) versus subclass B3 AIM-1 β-lactamase (PDB: 4AWZ[2]) (pink). Amino acid residues that are identical in sequence and position are labeled in black. Unique residues are labeled with the enzyme name and colored according to the structure. Note that AIM-1 residues Ser221 and Thr223 are positioned to replace the function of NDM-1 Lys224. Also, the side chain of AIM-1 Gln157 is positioned near that of NDM-1 Asn233. Finally, AIM-1 His121 serves as a zinc ligand in place of Cys221 of NDM-1, which is Ser221 in AIM-1. The zinc ions are shown in tan for NDM-1 and pink for AIM-1.

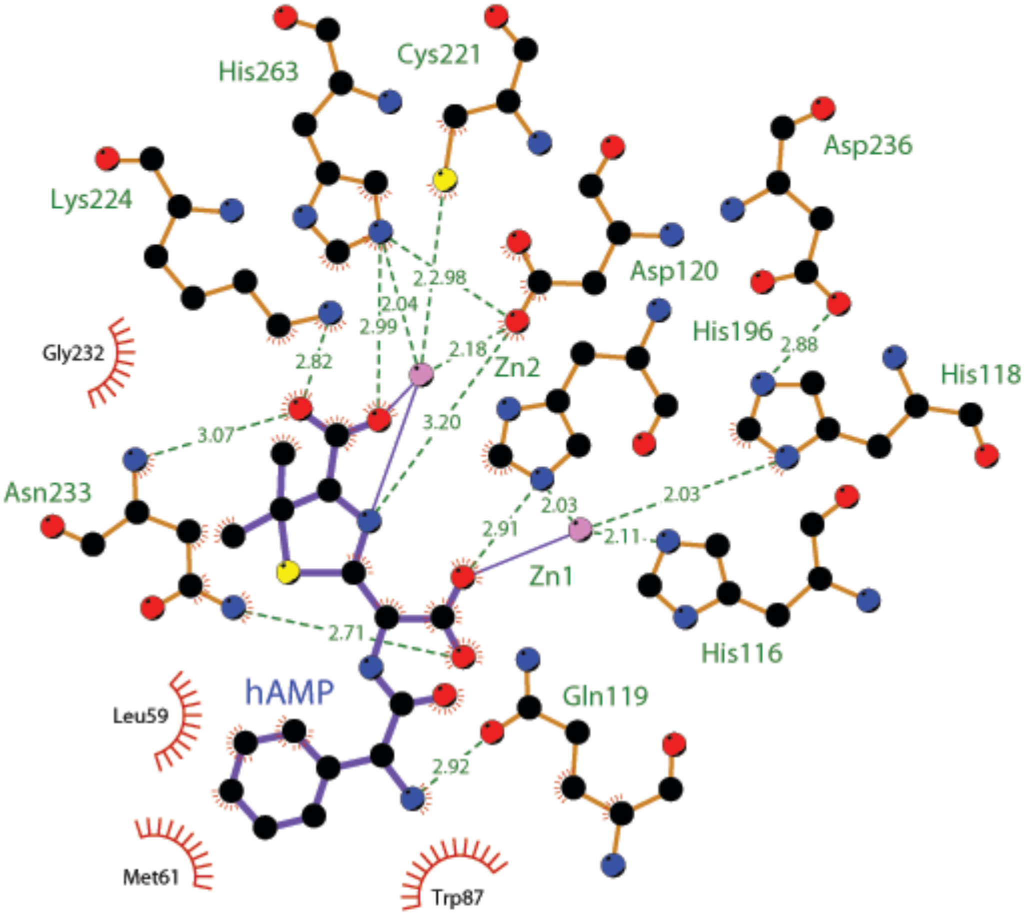

Supplementary Figure 9. Interactions between wild-type NDM-1 and hydrolyzed ampicillin. Ligand-protein and ligand-Zn^{2+} interactions in subunit A of NDM-1 in complex with hydrolyzed ampicillin (PDB: 3Q6X) [3] are depicted using LIGPLOT [4]. The hydrolytic product of ampicillin (hAMP) and NDM-1 active site residue are shown as purple and orange sticks, respectively, with atoms colored by type (C, black; N, blue; O, red; S, yellow). Hydrogen bonding interactions are shown as dashed green lines and the length of hydrogen bonds (Å) is labelled. Ligand-protein hydrophobic contacts are shown as red indented curves.

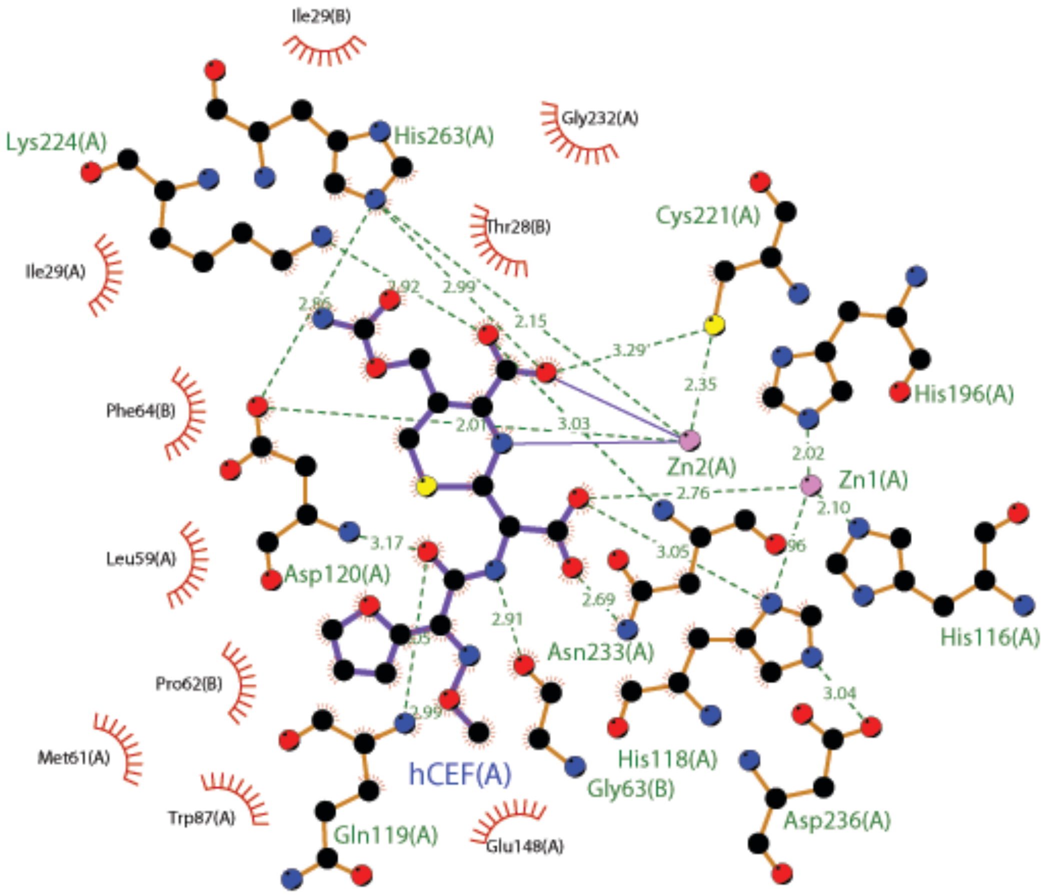

Supplementary Figure 10. Interactions between wild-type NDM-1 and hydrolyzed cefuroxime. Ligand-protein and ligand-Zn^{2+} interactions in subunit A of NDM-1 in complex with hydrolyzed cefuroxime (PDB: 4RL0) [5] depicted using LIGPLOT [4]. The hydrolytic product of cefuroxime (hCEF) and NDM-1 active site residue are shown as purple and orange sticks, respectively, with atoms colored by type (C, black; N, blue; O, red; S, yellow). Hydrogen bonding interactions are shown as dashed green lines and the length of hydrogen bonds (Å) is labelled. Ligand-protein hydrophobic contacts are shown as red indented curves. The residues in subunit B of NDM-1 forming hydrophobic interaction with hCEF are also shown.

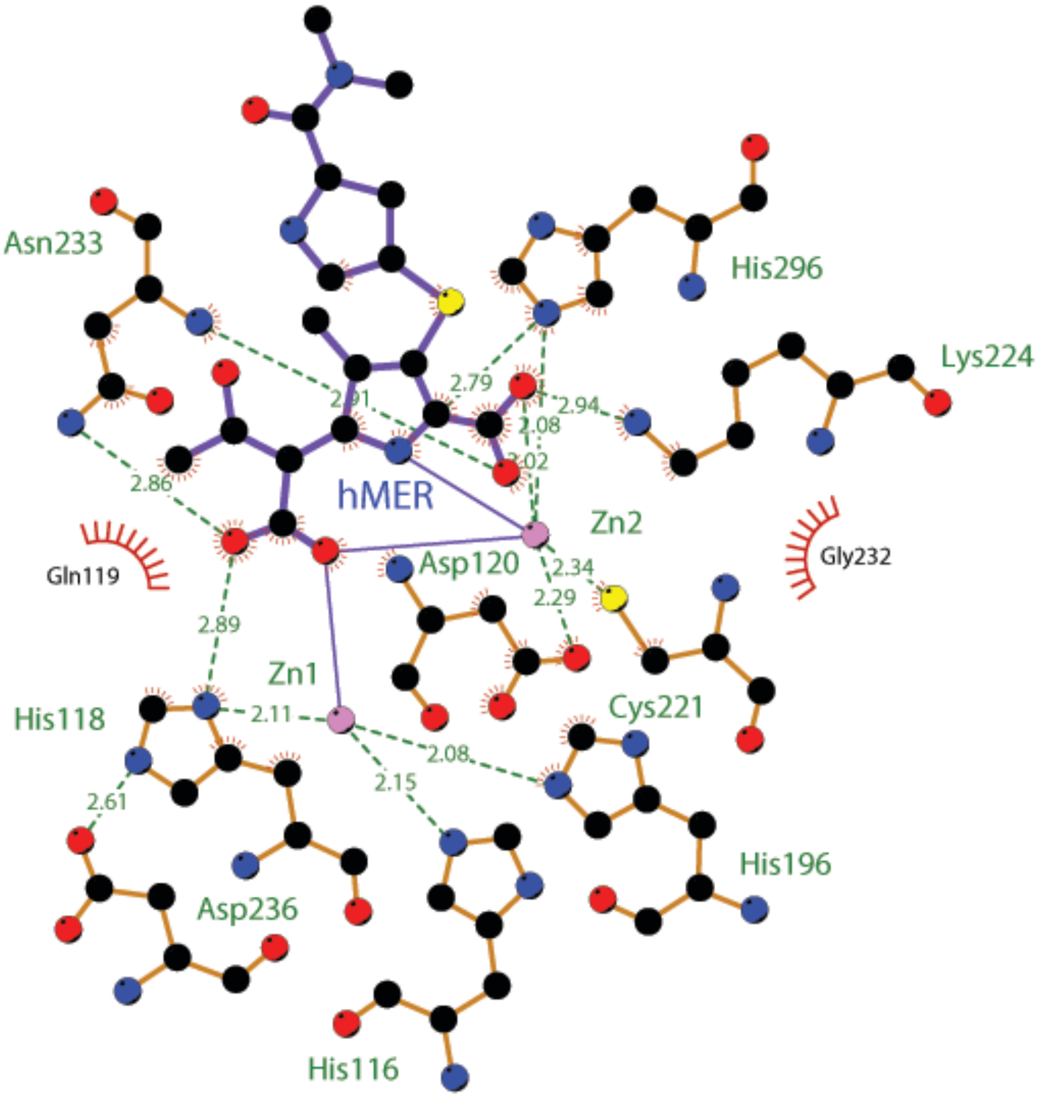

Supplementary Figure 11. Interactions between wild-type NDM-1 and hydrolyzed meropenem. Ligand-protein and ligand-Zn^{2+} interactions in subunit A of NDM-1 in complex with hydrolyzed meropenem (PDB: 4EYL) [6] depicted using LIGPLOT [4]. The hydrolytic product of meropenem (hMER) and NDM-1 active site residue are shown as purple and orange sticks, respectively, with atoms colored by type (C, black; N, blue; O, red; S, yellow). Hydrogen bonding interactions are shown as dashed green lines and the length of hydrogen bonds (Å) is labelled. Ligand-protein hydrophobic contacts are shown as red indented curves.

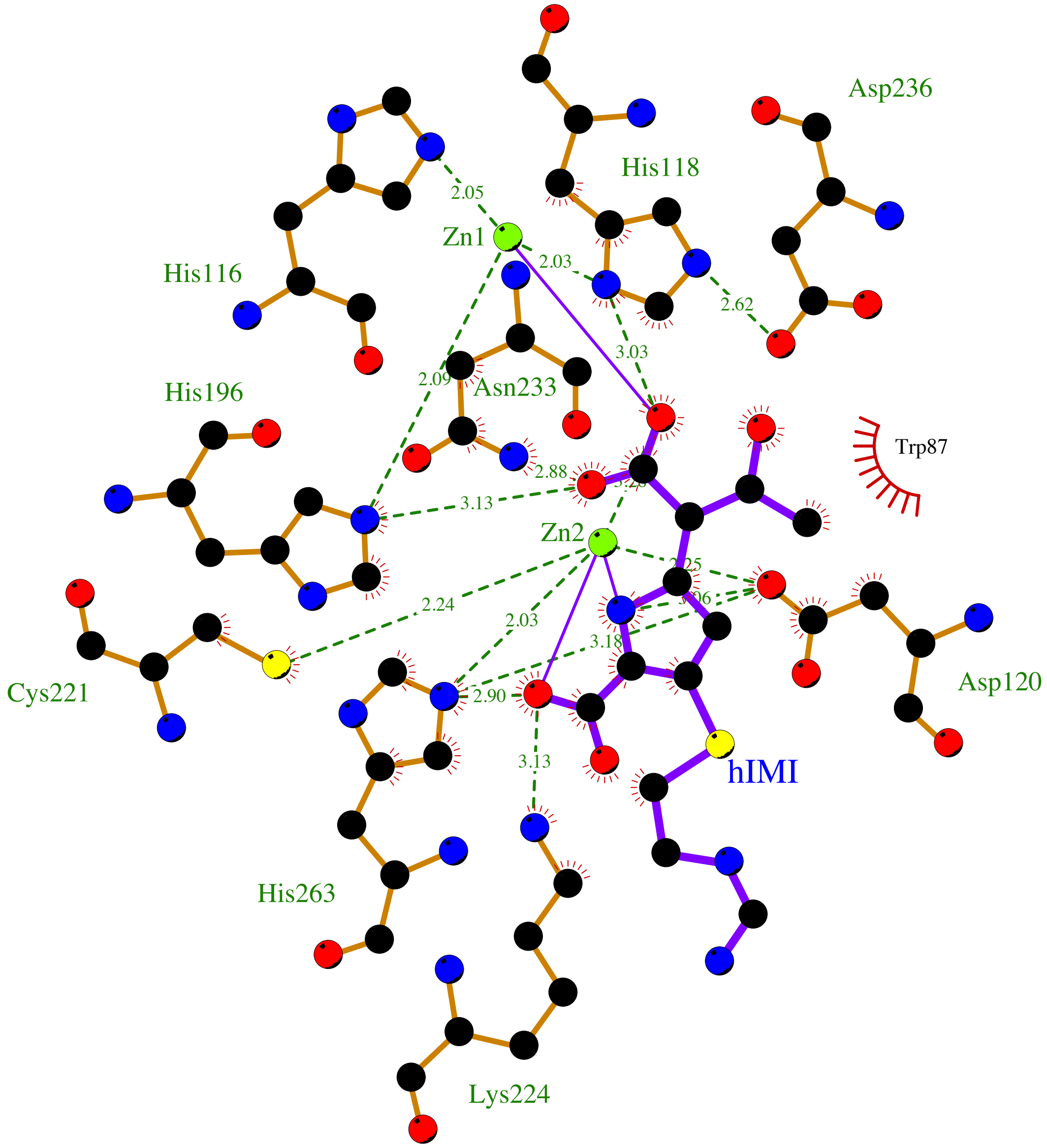

Supplementary Figure 12. Interactions between wild-type NDM-1 and hydrolyzed imipenem. Ligand-protein and ligand-Zn^{2+} interactions in subunit A of NDM-1 in complex with hydrolyzed imipenem (PDB: 5YPI) [6] depicted using LIGPLOT [4]. The hydrolytic product of imipenem (hIMI) and NDM-1 active site residues are shown as purple and orange sticks, respectively, with atoms colored by type (C, black; N, blue; O, red; S, yellow). Hydrogen bonding interactions are shown as dashed green lines and the length of hydrogen bonds (Å) is labelled. Ligand-protein hydrophobic contacts are shown as red indented curves.

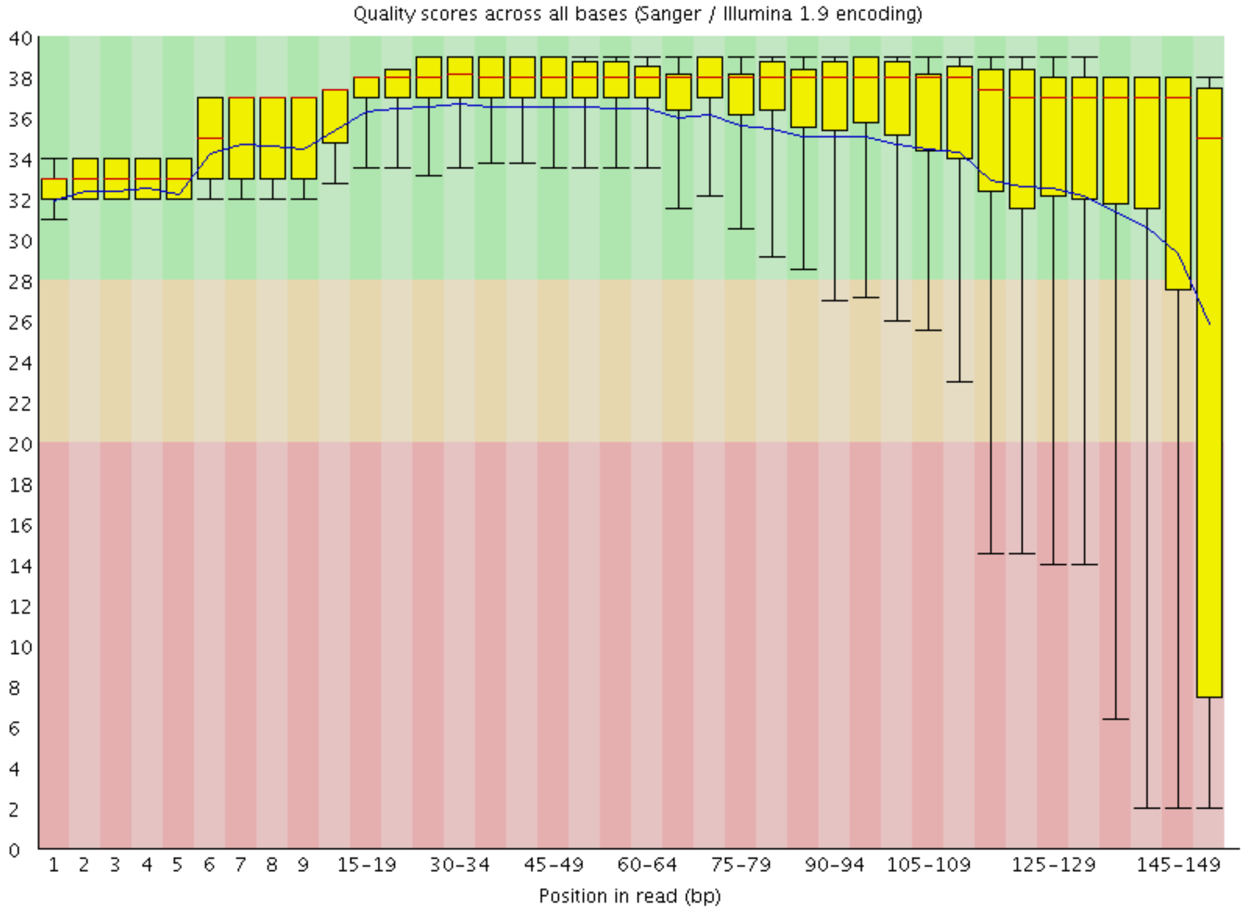

Supplementary Figure 13. Quality scores for Illumina DNA sequencing reads. The scores were generated with the Galaxy web server where the file containing sequencing data was uploaded onto https://usegalaxy.org/ and the FASTQ groomer was run to convert the FASTQ file to standard format. FastQC Read Quality reports were produced, which indicate per base sequence quality scores. The per base sequence quality is shown as a BoxWhisker type plot to indicate the average quality score at each position across all reads. In the graph, the x-axis shows the position in the sequence read and the y-axis shows the quality scores. Higher scores indicate higher confidence in the base call. The background of the graph divides the y axis into calls of very good quality (green), calls of reasonable quality (orange), and calls of poor quality (red). The yellow bars represent the inter-quartile range (25-75%) and the central red line represents the median value of the quality score. The upper and lower whiskers represent the 10% and 90% points of the quality scores, representatively. The blue line indicates the mean quality score.

Supplementary Table 1. Kinetic parameters for β-lactam hydrolysis

Enzyme	Kinetic parameters	Substrate[a]	
		Benzylpenicillin	Meropenem
NDM-1 wild-type	K_M (μM)	104 ± 15	37 ± 2
	k_{cat} (s^{-1})	739 ± 11	178 ± 0.6
	k_{cat} / K_M ($\mu M^{-1}s^{-1}$)	7.17 ± 0.95	4.79 ± 0.27
K224R/G232A/N233Q	K_M (μM)	843 ± 161	ND
	k_{cat} (s^{-1})	239 ± 30	ND
	k_{cat} / K_M ($\mu M^{-1}s^{-1}$)	0.29 ± 0.02	0.019 ± 0.0004

[a]Data are mean and standard deviations of at least two independent experiments; ND, not determined.

Supplementary Table 2. Primers used in this study

Primer name	Sequence (5' to 3')[a]
NDM SacI 5'	CGAGCTCATGGAATTGCCCAATATTATG
NDM StrepII XbaI 3'	GCTCTAGATCATTTTTCGAACTGCGGGTGGCTCCAAGCGCTGCGCAG CTTGTCGGCCATG
NDM NdeI 5'	CGCATATGGGCCAGCAAATGGAAACTG
NDM XhoI 3'	CTCTCGAGTCAGCGCAGCTTGTCGGC
NDM62XhoI For	ATCTCGACATCTCGAGCCGGGTTTCGGGGCAGTCGCTTC
NDM62XhoI Rev	GAAACCCGGCTCGAGATGTCGAGATAGGAAGTGTGCTG
NDM65XhoI For	GCCGGGTTTCTCGAGGGGCAGTCGCTTCCAACGGTTTG
NDM65XhoI Rev	GCGACTGCCCCTCGAGAAACCCGGCATGTCGAGATAGGAAG
NDM68XhoI For	GGCAGTCGCTCGAGTCCAACGGTTTGATCGTCAGGGATG
NDM68XhoI Rev	CAAACCGTTGGACTCGAGCGACTGCCCCGAAACCCGGCATG
NDM85XhoI For	GGTCGATACCTCGAGCCTGGACCGATGACCAGACCGC
NDM85XhoI Rev	TCGGTCCAGGCTCGAGGTATCGACCACCAGCACGCGGCC
NDM117XhoI For	GTGACTCACTCGAGCGCATCAGGACAAGATGGGCGG
NDM117XhoI Rev	TGTCCTGATGCGCTCGAGTGAGTCACCACCGCCAGCGCGAC
NDM120XhoI For	GCATCAGGACTCGAGAAGATGGGCGGTATGGACGCGC
NDM120XhoI Rev	CGCCCATCTTCTCGAGTCCTGATGCGCGTGAGTCACCAC
NDM195XhoI For	CCCGGCCCCGCTCGAGCCACACCAGTGACAATATCACC
NDM195XhoI Rev	CTGGTGTGGCTCGAGCGGGGCCGGGGTAAAATACCTTG
NDM198XhoI For	CGGCCACACCTCGAGAGTGACAATATCACCGTTGGGATC
NDM198XhoI Rev	TATTGTCACTCTCGAGGTGTGGCCGGGGCCGGGGTAA
NDM 220 XhoI For	CGCTTTTGGTCTCGAGGCTGCCTGATCAAGGACAGCAAG
NDM 220 XhoI Rev	GATCAGGCAGCCTCGAGACCAAAAGCGATGTCGGTGCCG
NDM223XhoI For	CTGCCTGATCTCGAGAAGGACAGCAAGGCCAAGTCGCTC
NDM223XhoI Rev	TGCTGTCCTTCTCGAGATCAGGCAGCCACCAAAAGCGATG
NDM228XhoI For	CAGCAAGGCCTCGAGAAGTCGCTCGGCAATCTCGGTGATG
NDM228XhoI Rev	CGAGCGACTTCTCGAGGCCTTGCTGTCCTTGATCAGGC
NDM232XhoI For	GTCGCTCGGCTCGAGAATCTCGGTGATGCCGACACTGAG
NDM232XhoI Rev	CACCGAGATTCTCGAGCCGAGCGACTTGGCCTTGCTGTC
NDM236XhoI For	CAATCTCGGTCTCGAGATGCCGACACTGAGCACTACGC
NDM236XhoI Rev	TGTCGGCATCTCGAGACCGAGATTGCCGAGCGACTTGG
NDM262XhoI For	GATCGTGATGCTCGAGCCATTCCGCCCCCGATAGCCG
NDM262XhoI Rev	GCGGAATGGCTCGAGCATCACGATCATGCTGGCCTTGG
NDM 61 libraryFor	CTATCTCGACNNSCCGGGTTTCGGGGCAGTCGCTTCC
NDM 61 libraryRev	CCCGAAACCCGGSNNGTCGAGATAGGAAGTGTGCTGCC
NDM 62 libraryFor	TCTCGACATGNNSGGTTTCGGGGCAGTCGCTTCCAAC
NDM 62 libraryRev	TGCCCCGAAACCSNNCATGTCGAGATAGGAAGTGTGC
NDM 63 libraryFor	CGACATGCCGNNSTTCGGGGCAGTCGCTTCCAACGGT
NDM 63 libraryRev	GACTGCCCCGAASNNCGGCATGTCGAGATAGGAAGTG

NDM 64 libraryFor	CATGCCGGGTNNSGGGGCAGTCGCTTCCAACGGTTTG
NDM 64 libraryRev	AGCGACTGCCCCSNNACCCGGCATGTCGAGATAGGAAG
NDM 65 libraryFor	GCCGGGTTTCNNSGCAGTCGCTTCCAACGGTTTGATC
NDM 65 libraryRev	GGAAGCGACTGCSNNGAAACCCGGCATGTCGAGATAG
NDM 67 libraryFor	TTTCGGGGCANNSGCTTCCAACGGTTTGATCGTCAGG
NDM 67 libraryRev	ACCGTTGGAAGCSNNTGCCCCGAAACCCGGCATGTCG
NDM 68 libraryFor	CGGGGCAGTCNNSTCCAACGGTTTGATCGTCAGGGAT
NDM 68 libraryRev	CAAACCGTTGGASNNGACTGCCCCGAAACCCGGCATG
NDM 69 libraryFor	GGCAGTCGCTNNSAACGGTTTGATCGTCAGGGATGGC
NDM 69 libraryRev	GATCAAACCGTTSNNAGCGACTGCCCCGAAACCCGGC
NDM 84 libraryFor	GCTGGTGGTCNNSACCGCCTGGACCGATGACCAGACC
NDM 84 libraryRev	GGTCCAGGCGGTSNNGACCACCAGCACGCGGCCGCCA
NDM 87 libraryFor	CGATACCGCCNNSACCGATGACCAGACCGCCCAGATC
NDM 87 libraryRev	CTGGTCATCGGTSNNGGCGGTATCGACCACCAGCACG
NDM 116 libraryFor	GGTGGTGACTNNSGCGCATCAGGACAAGATGGGCGGT
NDM 116 libraryRev	GTCCTGATGCGCSNNAGTCACCACCGCCAGCGCGACC
NDM 117 libraryFor	GGTGACTCACNNSCATCAGGACAAGATGGGCGGTATG
NDM 117 libraryRev	CTTGTCCTGATGSNNGTGAGTCACCACCGCCAGCGCG
NDM 118 libraryFor	GACTCACGCGNNSCAGGACAAGATGGGCGGTATGGAC
NDM 118 libraryRev	CATCTTGTCCTGSNNCGCGTGAGTCACCACCGCCAGC
NDM 120 libraryFor	CGCGCATCAGNNSAAGATGGGCGGTATGGACGCGCTG
NDM 120 libraryRev	ACCGCCCATCTTSNNCTGATGCGCGTGAGTCACCACC
NDM 121 libraryFor	GCATCAGGACNNSATGGGCGGTATGGACGCGCTGCAT
NDM 121 libraryRev	CATACCGCCCATSNNGTCCTGATGCGCGTGAGTCACC
NDM 194 libraryFor	TTACCCCGGCNNSGGCCACACCAGTGACAATATCACC
NDM 194 libraryRev	ACTGGTGTGGCCSNNGCCGGGGTAAAATACCTTGAGC
NDM 195 libraryFor	CCCCGGCCCCNNSCACACCAGTGACAATATCACCGTT
NDM 195 libraryRev	GTCACTGGTGTGSNNGGGGCCGGGGTAAAATACCTTG
NDM 196 libraryFor	CGGCCCCGGCNNSACCAGTGACAATATCACCGTTGGG
NDM 196 libraryRev	ATTGTCACTGGTSNNGCCGGGGCCGGGGTAAAATACC
NDM 197 library For	CCCCGGCCACNNSAGTGACAATATCACCGTTGGGATC
NDM 197 libraryRev	GATATTGTCACTSNNGTGGCCGGGGCCGGGGTAAAAT
NDM 198 libraryFor	CGGCCACACCNNSGACAATATCACCGTTGGGATCGAC
NDM 198 libraryRev	GGTGATATTGTCSNNGGTGTGGCCGGGGCCGGGGTAA
NDM 199 library For	CCACACCAGTNNSAATATCACCGTTGGGATCGACGGC
NDM 199 libraryRev	AACGGTGATATTSNNACTGGTGTGGCCGGGGCCGGGG
NDM 220 libraryFor	CGCTTTTGGTNNSTGCCTGATCAAGGACAGCAAGGCC
NDM 220 libraryRev	CTTGATCAGGCASNNACCAAAAGCGATGTCGGTGCCG
NDM 221 libraryFor	CTTTTGGTGGCNNSCTGATCAAGGACAGCAAGGCCAAG
NDM 221 libraryRev	GTCCTTGATCAGSNNGCCACCAAAAGCGATGTCGGTG
NDM 224 libraryFor	CTGCCTGATCNNSGACAGCAAGGCCAAGTCGCTCGGC
NDM 224 libraryRev	GGCCTTGCTGTCSNNGATCAGGCAGCCACCAAAAGCG
NDM 225 libraryFor	CCTGATCAAGNNSAGCAAGGCCAAGTCGCTCGGCAAT

NDM 225 libraryRev	CTTGGCCTTGCTSNNCTTGATCAGGCAGCCACCAAAAG
NDM 228 libraryFor	GGACAGCAAGNNSAAGTCGCTCGGCAATCTCGGTGAT
NDM 228 libraryRev	GCCGAGCGACTTSNNCTTGCTGTCCTTGATCAGGCAG
NDM 232 libraryFor	CAAGTCGCTCNNSAATCTCGGTGATGCCGACACTGAG
NDM 232 libraryRev	ATCACCGAGATTSNNGAGCGACTTGGCCTTGCTGTCC
NDM 233 libraryFor	GTCGCTCGGCNNSCTCGGTGATGCCGACACTGAGCAC
NDM 233 libraryRev	GGCATCACCGAGSNNGCCGAGCGACTTGGCCTTGCTG
NDM 236 libraryFor	CAATCTCGGTNNSGCCGACACTGAGCACTACGCCGCG
NDM 236 libraryRev	CTCAGTGTCGGCSNNACCGAGATTGCCGAGCGACTTG
NDM 262 libraryFor	GATCGTGATGNNSCATTCCGCCCCCGATAGCCGCGCC
NDM 262 libraryRev	GGGGGCGGAATGSNNCATCACGATCATGCTGGCCTTG
NDM 263 libraryFor	CGTGATGAGCNNSTCCGCCCCCGATAGCCGCGCCGCA
NDM 263 libraryRev	ATCGGGGGCGGASNNGCTCATCACGATCATGCTGGCC
NDM F64H For	CATGCCGGGTCACGGGGCAGTCGCTTCCAACGGTTTG
NDM F64H Rev	AGCGACTGCCCCGTGACCCGGCATGTCGAGATAGGAAG
NDM D84E For	GCTGGTGGTCGAGACCGCCTGGACCGATGACCAGACC
NDM D84E Rev	GGTCCAGGCGGTCTCGACCACCAGCACGCGGCCGCCA
NDM W87P For	CGATACCGCCCCGACCGATGACCAGACCGCCCAGATC
NDM W87P Rev	CTGGTCATCGGTCGGGGCGGTATCGACCACCAGCACG
NDM K121R For	GCATCAGGACCGCATGGGCGGTATGGACGCGCTGCAT
NDM K121R Rev	CATACCGCCCATGCGGTCCTGATGCGCGTGAGTCACC
NDM K121T For	GCATCAGGACACCATGGGCGGTATGGACGCGCTGCAT
NDM K121T Rev	CATACCGCCCATGGTGTCCTGATGCGCGTGAGTCACC
NDM G195A For	CCCCGGCCCCGCGCACACCAGTGACAATATCACCGTT
NDM G195A Rev	GTCACTGGTGTGCGCGGGGCCGGGGTAAAATACCTTG
NDM D199E For	CCACACCAGTGAGAATATCACCGTTGGGATCGACGGC
NDM D199E Rev	AACGGTGATATTCTCACTGGTGTGGCCGGGGCCGGGG
NDM K224R For	CTGCCTGATCCGCGACAGCAAGGCCAAGTCGCTCGGC
NDM K224R Rev	GGCCTTGCTGTCGCGGATCAGGCAGCCACCAAAAGCG
NDM K224H For	CTGCCTGATCCACGACAGCAAGGCCAAGTCGCTCGGC
NDM K224H Rev	GGCCTTGCTGTCGTGGATCAGGCAGCCACCAAAAGCG
NDM D225E For	CCTGATCAAGGAGAGCAAGGCCAAGTCGCTCGGCAAT
NDM D225E Rev	CTTGGCCTTGCTCTCCTTGATCAGGCAGCCACCAAAAG
NDM G232A For	CAAGTCGCTCGCGAATCTCGGTGATGCCGACACTGAG
NDM G232A Rev	ATCACCGAGATTCGCGAGCGACTTGGCCTTGCTGTCC
NDM N233H For	GTCGCTCGGCCACCTCGGTGATGCCGACACTGAGCAC
NDM N233H Rev	GGCATCACCGAGGTGGCCGAGCGACTTGGCCTTGCTG
NDM N233Q For	GTCGCTCGGCCAGCTCGGTGATGCCGACACTGAGCAC
NDM N233Q Rev	GGCATCACCGAGCTGGCCGAGCGACTTGGCCTTGCTG
NDM G232AN233Q For	CAAGTCGCTCGCGCAGCTCGGTGATGCCGACACTGAG
NDM G232AN233Q Rev	ATCACCGAGCTGCGCGAGCGACTTGGCCTTGCTGTCC

NDM61Naive	NKKNKKCCTGCGACACACTTCCTATCTCGAC
NDM61Amp	NKKNKKTGCAGAGCACACTTCCTATCTCGAC
NDM61CT	NKKNKKACCTAGGCACACTTCCTATCTCGAC
NDM61IMP	NKKNKKTTGATCCCACACTTCCTATCTCGAC
NDM62Naive	NKKNKKATCTTGCACTTCCTATCTCGACATG
NDM62Amp	NKKNKKTCTCCATACTTCCTATCTCGACATG
NDM62CT	NKKNKKCATCGAGACTTCCTATCTCGACATG
NDM62IMP	NKKNKKTTCGAGCACTTCCTATCTCGACATG
NDM63Naive	NKKNKKAGTTGGTTCCTATCTCGACATGCCG
NDM63Amp	NKKNKKGTACCGGTCCTATCTCGACATGCCG
NDM63CT	NKKNKKCGGAGTTTCCTATCTCGACATGCCG
NDM63IMP	NKKNKKACTTCAATCCTATCTCGACATGCCG
NDM64Naive	NKKNKKTGATAGTTATCTCGACATGCCGGGT
NDM64Amp	NKKNKKGATCCAATATCTCGACATGCCGGGT
NDM64CT	NKKNKKCAGGTCGTATCTCGACATGCCGGGT
NDM64IMP	NKKNKKCGCATTATATCTCGACATGCCGGGT
NDM61to64R	TGAGGATCTGGGCGGTCT
NDM65Naive	NKKNKKGGTACCTCTCGACATGCCGGGTTTC
NDM65Amp	NKKNKKGGACGCACTCGACATGCCGGGTTTC
NDM65CT	NKKNKKGAGATTCCTCGACATGCCGGGTTTC
NDM65IMP	NKKNKKGAGCATGCTCGACATGCCGGGTTTC
NDM67Naive	NKKNKKGTTGCGTATGCCGGGTTTCGGGGCA
NDM67Amp	NKKNKKCCAATGCATGCCGGGTTTCGGGGCA
NDM67CT	NKKNKKCGAGATCATGCCGGGTTTCGGGGCA
NDM67IMP	NKKNKKCATATTGATGCCGGGTTTCGGGGCA
NDM68Naive	NKKNKKGACGTCACCGGGTTTCGGGGCAGTC
NDM68Amp	NKKNKKTGGCATCCCGGGTTTCGGGGCAGTC
NDM68CT	NKKNKKGTAATTGCCGGGTTTCGGGGCAGTC
NDM68IMP	NKKNKKCCTATCTCCGGGTTTCGGGGCAGTC
NDM69Naive	NKKNKKCAATCGGGGTTTCGGGGCAGTCGCT
NDM69Amp	NKKNKKGCGGCATGGTTTCGGGGCAGTCGCT
NDM69CT	NKKNKKAGTACTGGGTTTCGGGGCAGTCGCT
NDM69IMP	NKKNKKTACTATTGGTTTCGGGGCAGTCGCT
NDM65to69R	GCTTGATCCAGTTGAGGATCTG
NDM84Naive	NKKNKKCCGGATGGGCCGCGTGCTGGTGGTC
NDM84Amp	NKKNKKACCATGAGGCCGCGTGCTGGTGGTC
NDM84CT	NKKNKKCGGTTCTGGCCGCGTGCTGGTGGTC
NDM84IMP	NKKNKKTATTCCAGGCCGCGTGCTGGTGGTC
NDM87Naive	NKKNKKCCTCCTGCTGGTGGTCGATACCGCC
NDM87Amp	NKKNKKAGGTATTCTGGTGGTCGATACCGCC
NDM87CT	NKKNKKGCATTCGCTGGTGGTCGATACCGCC
NDM87IMP	NKKNKKTTGCGAACTGGTGGTCGATACCGCC
NDM84to87R	TCTTGTCCTGATGCGCGTG

NDM116Naive	NKKNKKTTGAATTGCGCTGGCGGTGGTGACT
NDM116Amp	NKKNKKCTGCGCGGCGCTGGCGGTGGTGACT
NDM116CT	NKKNKKAGACCTTGCGCTGGCGGTGGTGACT
NDM116IMP	NKKNKKGTCCAGTGCGCTGGCGGTGGTGACT
NDM117Naive	NKKNKKACCTGCTCTGGCGGTGGTGACTCAC
NDM117Amp	NKKNKKCCGGTACCTGGCGGTGGTGACTCAC
NDM117CT	NKKNKKCTTGACCCTGGCGGTGGTGACTCAC
NDM117IMP	NKKNKKCATCATTCTGGCGGTGGTGACTCAC
NDM118Naive	NKKNKKTCTGACTGCGGTGGTGACTCACGCG
NDM118Amp	NKKNKKTCTAGTTGCGGTGGTGACTCACGCG
NDM118CT	NKKNKKGCCATAGGCGGTGGTGACTCACGCG
NDM118IMP	NKKNKKACCGTCGGCGGTGGTGACTCACGCG
NDM116to118R	CAACCATCCCCTCTTGCG
NDM120Naive	NKKNKKCTTGGTTGTGACTCACGCGCATCAG
NDM120Amp	NKKNKKTACGCCGGTGACTCACGCGCATCAG
NDM120CT	NKKNKKGGACTGCGTGACTCACGCGCATCAG
NDM120IMP	NKKNKKGCGCGAGGTGACTCACGCGCATCAG
NDM121Naive	NKKNKKGTCGCAGACTCACGCGCATCAGGAC
NDM121Amp	NKKNKKCATACGTACTCACGCGCATCAGGAC
NDM121CT	NKKNKKTCAGTATACTCACGCGCATCAGGAC
NDM121IMP	NKKNKKCTAAGTAACTCACGCGCATCAGGAC
NDM120to121R	AAGTCAGGCTGTGTTGCG
NDM194Naive	NKKNKKTTAGCTTAAGGTATTTTACCCCGGC
NDM194Amp	NKKNKKCGCCGTCAAGGTATTTTACCCCGGC
NDM194CT	NKKNKKGTCTTCTAAGGTATTTTACCCCGGC
NDM194IMP	NKKNKKGCCGGACAAGGTATTTTACCCCGGC
NDM195Naive	NKKNKKAAGCTGAGTATTTTACCCCGGCCCC
NDM195Amp	NKKNKKGCGCTCTGTATTTTACCCCGGCCCC
NDM195CT	NKKNKKCGTAGGCGTATTTTACCCCGGCCCC
NDM195IMP	NKKNKKATGATTAGTATTTTACCCCGGCCCC
NDM196Naive	NKKNKKGCAGGTTTTTACCCCGGCCCCGGC
NDM196Amp	NKKNKKAATCGTCTTTACCCCGGCCCCGGC
NDM196CT	NKKNKKCGGCCTATTTACCCCGGCCCCGGC
NDM196IMP	NKKNKKCTATGCCTTTACCCCGGCCCCGGC
NDM194to196R	CACCGAGATTGCCGAGCG
NDM197Naive	NKKNKKGGTTGAATACCCCGGCCCCGGCCAC
NDM197Amp	NKKNKKGAGTTAATACCCCGGCCCCGGCCAC
NDM197CT	NKKNKKTAGACTATACCCCGGCCCCGGCCAC
NDM197IMP	NKKNKKTCATGCATACCCCGGCCCCGGCCAC
NDM198Naive	NKKNKKGCTTATTCCCGGCCCCGGCCACACC
NDM198Amp	NKKNKKCAAGGCTCCCGGCCCCGGCCACACC
NDM198CT	NKKNKKAGGTTGGCCCGGCCCCGGCCACACC
NDM198IMP	NKKNKKCTTCTGCCCCGGCCCCGGCCACACC

NDM199Naive	NKKNKKTAATTCTGGCCCCGGCCACACCAGT
NDM199Amp	NKKNKKGATGCTGGGCCCCGGCCACACCAGT
NDM199CT	NKKNKKCCTAGAAGGCCCCGGCCACACCAGT
NDM199IMP	NKKNKKCTAGAGGGGCCCCGGCCACACCAGT
NDM197to199R	CATCACCGAGATTGCCGAG
NDM221Naive	NKKNKKTATCCGGGACATCGCTTTTGGTGGC
NDM221Amp	NKKNKKAGGCGGCGACATCGCTTTTGGTGGC
NDM221CT	NKKNKKGGTCGTTGACATCGCTTTTGGTGGC
NDM221IMP	NKKNKKCCGCTGGGACATCGCTTTTGGTGGC
NDM224Naive	NKKNKKGGAACTATTTGGTGGCTGCCTGATC
NDM224Amp	NKKNKKATTGCCATTTGGTGGCTGCCTGATC
NDM224CT	NKKNKKATATACGTTTGGTGGCTGCCTGATC
NDM224IMP	NKKNKKGATTAGCTTTGGTGGCTGCCTGATC
NDM225Naive	NKKNKKAGAAGTCGGTGGCTGCCTGATCAAG
NDM225Amp	NKKNKKATAGTACGGTGGCTGCCTGATCAAG
NDM225CT	NKKNKKGATCTCGGGTGGCTGCCTGATCAAG
NDM225IMP	NKKNKKGGCTGCGGGTGGCTGCCTGATCAAG
NDM221to225R	CATGCTGGCCTTGGGGAA
NDM228Naive	NKKNKKGCCTCTCCTGATCAAGGACAGCAAG
NDM228Amp	NKKNKKCTGAAGCCTGATCAAGGACAGCAAG
NDM228CT	NKKNKKTGGATATCTGATCAAGGACAGCAAG
NDM228IMP	NKKNKKGTATAACCTGATCAAGGACAGCAAG
NDM228R	CGGAATGGCTCATCACGATC
NDM232Naive	NKKNKKATTAAGGAGCAAGGCCAAGTCGCTC
NDM232Amp	NKKNKKGTTGGCGAGCAAGGCCAAGTCGCTC
NDM232CT	NKKNKKGCGAGTAAGCAAGGCCAAGTCGCTC
NDM232IMP	NKKNKKTGGCGCTAGCAAGGCCAAGTCGCTC
NDM233Naive	NKKNKKTTACTTAAAGGCCAAGTCGCTCGGC
NDM233Amp	NKKNKKTGACCAAAAGGCCAAGTCGCTCGGC
NDM233CT	NKKNKKATGCAACAAGGCCAAGTCGCTCGGC
NDM233IMP	NKKNKKAGAGGATAAGGCCAAGTCGCTCGGC
NDM236Naive	NKKNKKGACCGCCTCGCTCGGCAATCTCGGT
NDM236Amp	NKKNKKATCATATTCGCTCGGCAATCTCGGT
NDM236CT	NKKNKKGCGTACCTCGCTCGGCAATCTCGGT
NDM236IMP	NKKNKKCGTTCTTTCGCTCGGCAATCTCGGT
NDM232to236R	CTATCGGGGGCGGAATGG
NDM262Naive	NKKNKKTCAACGGGCCAGCATGATCGTGATG
NDM262Amp	NKKNKKGATTGCTGCCAGCATGATCGTGATG
NDM262CT	NKKNKKACGGAGCGCCAGCATGATCGTGATG
NDM262IMP	NKKNKKAGAGTTGGCCAGCATGATCGTGATG
NDM263Naive	NKKNKKGGAGTCTAGCATGATCGTGATGAGC
NDM263Amp	NKKNKKAAGATAGAGCATGATCGTGATGAGC
NDM263CT	NKKNKKACTGCTTAGCATGATCGTGATGAGC

NDM263IMP	NKKNKKCGGTAACAGCATGATCGTGATGAGC
NDM262to263R	GGGCTTGCGACTCTAGATC
NDM220Naive	NKKNKKTATCCGGACCGACATCGCTTTTGGT
NDM220Amp	NKKNKKAGGCGGCACCGACATCGCTTTTGGT
NDM220CT	NKKNKKGGTCGTTACCGACATCGCTTTTGGT
NDM220IMP	NKKNKKCCGCTGGACCGACATCGCTTTTGGT
NDM220R	GATCATGCTGGCCTTGGG

[a] N represents a mix of the 4 nucleotides, S indicates G and C, and K indicates G and T

Supplementary References

1. Sun Z, Mehta SC, Adamski CJ, Gibbs RA, Palzkill T. Deep sequencing of random mutant libraries reveals the active site of the narrow specificity CphA metallo-beta-lactamase is fragile to mutations. *Sci Rep* **6**, 33195 (2016).

2. Leiros H-KS*, et al.* Crystal structure of the mobile metallo-beta-lactamase AIM-1 from *Pseudomonas aeruginosa*: Insights into antibiotic binding and the role of Gln157. *Antimicrob Agents Chemother* **56**, 4341-4353 (2012).

3. Zhang H, Hao Q. Crystal structure of NDM-1 reveals a common beta-lactam hydrolysis mechanism. *FASEB J* **25**, 2574-2582 (2011).

4. Laskowski RA, Swindells MB. LigPlot+: multiple ligand-protein interaction diagrams for drug discovery. *J Chem Inf Model* **51**, 2778-2786 (2011).

5. Feng H*, et al.* Structural and mechanistic insights into NDM-1 catalyzed hydrolysis of cephalosporins. *J Am Chem Soc* **136**, 14694-14697 (2014).

6. King DT, Worrall LJ, Gruninger R, Strynadka NC. New Delhi metallo-beta-lactamase: structural insights into beta-lactam recognition and inhibition. *J Am Chem Soc* **134**, 11362-11365 (2012).
