# Supplementary material for: Differential active site requirements for NDM-1 β-lactamase hydrolysis of carbapenem versus penicillin and cephalosporin antibiotics
Source: Nat Commun. 2018 Oct 30;9:4524. doi: 10.1038/s41467-018-06839-1 (PMC6207675; doi:10.1038/s41467-018-06839-1)
Supplement: Supplementary file 3 — Description of Additional Supplementary Files [file 41467_2018_6839_MOESM3_ESM.docx]

**Description of Additional Supplementary Files**

File Name: Supplementary Data 1

Description: Amino acid occurrences in naïve and antibiotic-selected libraries. The number of occurrences of each amino acid type at each randomized position in the naïve and antibiotic-selected libraries of NDM-1 is shown. Wild-type amino acid types are labelled red in the corresponding position.
